# Effects of Adherence to a Higher Protein Diet on Weight Loss, Markers of Health, and Functional Capacity in Older Women Participating in a Resistance-Based Exercise Program

**DOI:** 10.3390/nu10081070

**Published:** 2018-08-11

**Authors:** Melyn Galbreath, Bill Campbell, Paul La Bounty, Jennifer Bunn, Jacqueline Dove, Travis Harvey, Geoffrey Hudson, Jean L. Gutierrez, Kyle Levers, Elfego Galvan, Andrew Jagim, Lori Greenwood, Matthew B. Cooke, Mike Greenwood, Chris Rasmussen, Richard B. Kreider

**Affiliations:** 1 Matrix Medical Net, New York, NY 10011, USA; galbreathmelyn@gmail.com; 2 Exercise Science Program, University of South Florida, Tampa, FL 33620, USA; bcampbell@usf.edu; 3 Exercise and Sports Science Department, University of Mary Hardin-Baylor, Belton, TX 76513, USA; plabounty@umhb.edu; 4 Department of Physical Therapy, Campbell University, Buies Creek, NC 27506, USA; bunnj@campbell.edu; 5 Department of Health, Human Performance and Recreation, Baylor University, Waco, TX 76798, USA; Jacqueline_Dove@baylor.edu; 6 United States Special Operations Command, Preservation of the Force and Family, Human Performance, MacDill AFB, Tampa, FL 33621, USA; Travis.Harvey@socom.mil; 7 Department of Health, Kinesiology, & Sport, The University of South Alabama, Mobile, AL 36688, USA; ghudson@southalabama.edu; 8 Department of Nutrition & Metabolism, University of Texas Medical Branch, Galveston, TX 77555, USA; jeanguti@utmb.edu; 9 Department of Exercise and Nutrition Sciences, George Washington University, Washington, DC 20037, USA; klevers@gwu.edu; 10 Division of Rehabilitation Sciences, University of Texas Medical Branch, Galveston, TX 77555, USA; fego.galvan@gmail.com; 11 School of Health Sciences, Lindenwood University, Saint Charles, MO 63301, USA; AJagim@lindenwood.edu; 12 Exercise & Sport Nutrition Lab, Human Clinical Research Facility, Department of Health and Kinesiology, Texas A & M University, College Station, TX 77843, USA; lori.greenwood@tamu.edu (L.G.); mgreenwood26@tamu.edu (M.G.); crasmussen@tamu.edu (C.R.); 13 Faculty of Health, Arts and Design, Swinburne University of Technology, Melbourne, VIC 3000, Australia; mbcooke@swin.edu.au

**Keywords:** diet, exercise, sarcopenia, functional capacity, elderly

## Abstract

Resistance training and maintenance of a higher protein diet have been recommended to help older individuals maintain muscle mass. This study examined whether adherence to a higher protein diet while participating in a resistance-based exercise program promoted more favorable changes in body composition, markers of health, and/or functional capacity in older females in comparison to following a traditional higher carbohydrate diet or exercise training alone with no diet intervention. In total, 54 overweight and obese females (65.9 ± 4.7 years; 78.7 ± 11 kg, 30.5 ± 4.1 kg/m^2^, 43.5 ± 3.6% fat) were randomly assigned to an exercise-only group (E), an exercise plus hypo-energetic higher carbohydrate (HC) diet, or a higher protein diet (HP) diet. Participants followed their respective diet plans and performed a supervised 30-min circuit-style resistance exercise program 3 d/wk. Participants were tested at 0, 10, and 14 weeks. Data were analyzed using univariate, multivariate, and repeated measures general linear model (GLM) statistics as well as one-way analysis of variance (ANOVA) of changes from baseline with [95% confidence intervals]. Results revealed that after 14 weeks, participants in the HP group experienced significantly greater reductions in weight (E −1.3 ± 2.3, [−2.4, −0.2]; HC −3.0 ± 3.1 [−4.5, −1.5]; HP −4.8 ± 3.2, [−6.4, −3.1]%, *p* = 0.003), fat mass (E −2.7 ± 3.8, [−4.6, −0.9]; HC −5.9 ± 4.2 [−8.0, −3.9]; HP −10.2 ± 5.8 [−13.2, –7.2%], *p* < 0.001), and body fat percentage (E −2.0 ± 3.5 [−3.7, −0.3]; HC −4.3 ± 3.2 [−5.9, −2.8]; HP −6.3 ± 3.5 [−8.1, −4.5] %, *p* = 0.002) with no significant reductions in fat-free mass or resting energy expenditure over time or among groups. Significant differences were observed in leptin (E −1.8 ± 34 [−18, 14]; HC 43.8 ± 55 [CI 16, 71]; HP −26.5 ± 70 [−63, −9.6] ng/mL, *p* = 0.001) and adiponectin (E 43.1 ± 76.2 [6.3, 79.8]; HC −27.9 ± 33.4 [−44.5, −11.3]; HP 52.3 ± 79 [11.9, 92.8] µg/mL, *p* = 0.001). All groups experienced significant improvements in muscular strength, muscular endurance, aerobic capacity, markers of balance and functional capacity, and several markers of health. These findings indicate that a higher protein diet while participating in a resistance-based exercise program promoted more favorable changes in body composition compared to a higher carbohydrate diet in older females.

## 1. Introduction

Obesity is considered the leading cause of preventable death world-wide and is associated with a myriad of medical co-morbidities, including diabetes, arthritis, pulmonary abnormalities, urinary incontinence, cataracts, and certain types of cancer [1,2,3,4]. Obesity complicates the aging process [1,5,6,7] particularly when associated with sarcopenic obesity [2,3,4,5,6,7,8,9,10,11,12]. The fastest growing age group in the United States are individuals over the age of 65 [13,14,15]. According to the Center for Disease Control (CDC)’s 2007–2010 National Health and Nutrition Examination Survey, 41% of individuals in the United States aged 65–74 years old and 28% of those above age 75 were obese [16]. Aging women have been shown to be less active, and more likely to suffer from falls and hip fractures, which may subsequently lead to increased health care expenditures [17,18]. Consequently, identifying strategies to promote effective weight and fat loss while maintaining muscle mass, strength, and functional capacity as women age can have significant public health implications. 

Numerous studies indicate that resistance exercise training can maintain and/or increase fat-free mass as one ages [19,20,21,22]. Accordingly, the American College of Sports Medicine (ACSM) recommends that elderly individuals engage in regular physical activity that includes resistance-exercise of all major muscle groups [23,24]. Additionally, it has been recommended that older individuals should increase dietary protein intake in an attempt to maintain muscle mass and prevent sarcopenia [25,26,27,28]. Together, these two lifestyle modifications are likely to offer synergistic benefits pertaining to body composition and muscular strength in elderly populations. We have previously reported that adherence to a circuit-style resistance exercise program and adherence to a higher protein hypo-energetic diet promoted greater weight and fat loss while maintaining muscle mass and preserving resting energy expenditure in pre-menopausal and post-menopausal women under the age of 55 [29,30,31,32,33,34,35]. Moreover, this program promoted gains in aerobic capacity, muscular strength, and endurance, and improved markers of metabolic syndrome [29,30,31,32,33,34,35]. Theoretically, incorporating resistance exercise while maintaining a higher protein hypo-energetic diet may promote more favorable changes in body composition in older individuals attempting to lose weight compared to adherence to a higher carbohydrate diet. The purpose of this study was to determine if this exercise and diet intervention strategy could be an effective way to promote weight and fat loss, maintain fat-free mass and resting energy expenditure, and/or improve health and fitness in older sedentary and overweight and obese women in comparison to following a traditional higher carbohydrate hypo-energetic diet or resistance exercise training alone.

## 2. Methods

### 2.1. Experimental Design

This study was conducted at a university-based research setting as a randomized, parallel, prospective diet and exercise intervention trial. Females between the ages of 60 and 75 years were randomly assigned to one of three experimental groups: no diet intervention + exercise (E); higher carbohydrate diet + exercise (HC); or higher protein diet + exercise (HP). Participants partook in the Curves^®^ (Curves International, Waco, TX, USA) fitness and/or weight management program for 14 weeks. Those assigned to the diet interventions were assigned similar hypo-energetic diets comprised of higher carbohydrate or higher protein macronutrient distributions. Primary outcome variables included body weight, body composition (i.e., fat mass, fat free mass, percent body fat), and resting energy expenditure. Secondary outcome variables included resting hemodynamics, aerobic capacity, muscular strength, muscular endurance, functional capacity and balance, metabolic and appetite-related hormones, and clinical blood panels. 

### 2.2. Participants

This research protocol was approved by a university internal review board for the protection of human participants prior to initiation. Participants were recruited from local newspapers, radio advertisements, flyers, and the Internet. Participants meeting eligibility criteria attended a familiarization session. Entrance criteria stipulated recruitment of sedentary females between the ages of 60–75 years with a body mass index (BMI) greater than 27 kg/m^2^ and/or body fat percentage above 35% and no recent participation in a diet or exercise program or weight loss within the previous six months. Participants obtained medical clearance from their family physician prior to participating in baseline assessments. Exclusion criteria included: (1) presence of uncontrolled metabolic disorders including known electrolyte abnormalities, heart disease, arrhythmias, diabetes, or thyroid disease; (2) history of hypertension or hepatorenal, musculoskeletal, autoimmune, or neurological diseases; (3) current prescription of thyroid, hyperlipidemic, hypoglycemic, anti-hypertensive, or androgenic medications; or (4) consumption of ergogenic levels of nutritional supplements that may affect muscle mass (e.g., creatine, β-Hydroxy β-methylbutyric acid, anabolic/catabolic hormone levels (e.g., dehydroepiandrosterone), or weight loss (e.g., thermogenics) within the three months prior to the start of the study. 

**Figure 1:**
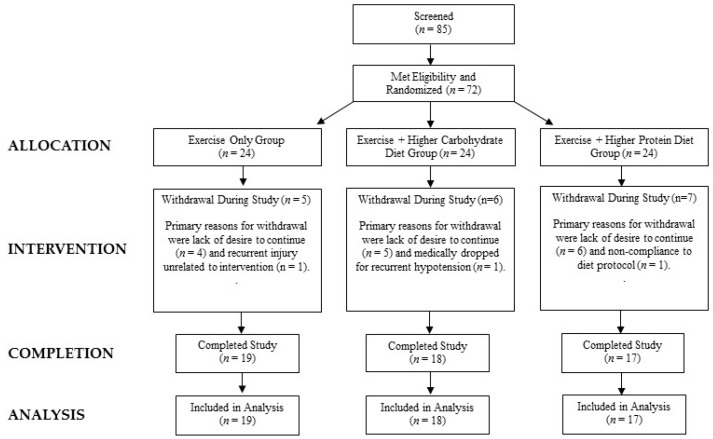
Consolidated standards of reporting trials (CONSORT) flow chart.

Figure 1 presents a consolidated standards of reporting trials (CONSORT) diagram. A total of 72 women met the entrance criteria, completed baseline testing, and began the exercise and/or diet program intervention. Fifteen participants withdrew during the first month citing a lack of desire to continue. One participant had recurrent musculoskeletal complaints unrelated to the intervention and was dropped during the second month. One participant was dropped for medical reasons during the last week of the study due to recurrent symptoms of hypotension n, and one participant was dropped due to failure to comply with dietary instructions and/or training compliance. Therefore, a total of 54 volunteers completed the 14-week intervention and were included in the analysis. This included 17 participants in the higher protein (HP) group, 18 participants the higher carbohydrate group (HC), and 19 participants in the exercise-only (E) group.

### 2.3. Testing Sequence

**Figure 2:**
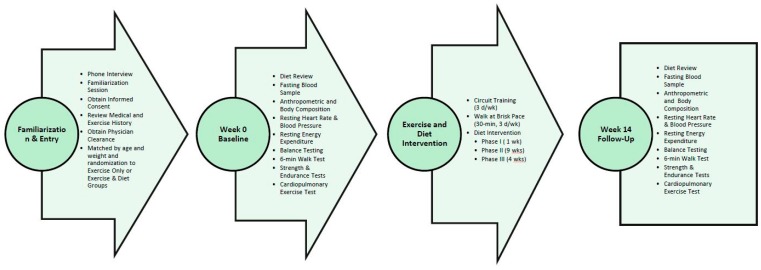
Overview of study testing and timeline.

Figure 2 provides an overview of the experimental design. Participants attended a detailed familiarization session prior to baseline testing. Body composition and clinical assessments were obtained at 0, 10, and 14 weeks. Dietary records (three weekdays and one weekend day) were obtained prior to each testing session. Participants were asked to refrain from vigorous physical activity, alcohol intake, and ingestion of over the counter medications for 24 h prior to testing. In addition, participants fasted for 12 h prior to reporting to the laboratory. All testing was conducted in the early morning hours to control for diurnal variations in hormone levels. The following measures were obtained at each testing session: weight; total body water determined by bioelectrical impedance (BIA); body composition determined by dual energy X-ray absorptiometry (DEXA); hip and waist measurements; resting energy expenditure (REE); resting blood pressure measurement; fasting whole blood and serum samples; balance tests that included the Sensory Organization Test (SOT), Limits of Stability (LOS), Step Up and Over (SUO), and Sit to Stand (STS) assessments; 1 repetition maximum (1RM) lifts on the bench press and leg press; upper and lower body muscular endurance (maximum repetitions performed at 80% of 1RM); a 6-min walk test; and a maximal cardiopulmonary exercise stress test. Participants also completed a medical safety and side effect report that was analyzed by the lab research nurse weekly.

### 2.4. Diet Intervention

Participants were assigned to one of the following three groups: (1) a no diet + exercise only (E); (2) a higher carbohydrate diet + exercise (HC); or (3) a higher protein diet + exercise (HP). Participants assigned to the no diet + exercise only control group participated in training sessions while maintaining their normal dietary habits. Participants assigned to the weight loss diet interventions followed Curves weight loss program. They were isocaloric and macronutrient content followed methods previously described [29,30,31,32,33,34,35]. In both dietary interventions, participants were assigned diets consisting of 1200 kcal/d for 1 week (Phase I) followed by 1600 kcal/d for 9-weeks (Phase II) over the course of a 10-week active weight loss period. The HC diet followed a traditional higher carbohydrate/low fat diet (i.e., 55% carbohydrate, 15% protein, and 30% fat). The HP diet offered higher protein food options with a goal of increasing dietary protein to approximately 1.2 g/kg/d and reducing carbohydrate intake while maintaining 30% of calories from fat. The final 4 weeks (Phase III) served as a weight maintenance period in which participants in both diet groups were given traditional higher carbohydrate diets with a goal of ingesting about 2100 kcal/d. Participants were instructed to monitor their body weight daily and to follow their assigned 1200 kcal/d diet for 2 d if they gained 3 lbs. of weight. Participants were given dietary plans and meal menus to follow at the start of the study. Participants met with a registered dietitian and/or exercise physiologist every two weeks and during each testing session to discuss diet and exercise compliance. Previous research in our lab has demonstrated that this diet intervention generally increases protein intake to 30% to 40% while maintaining fat intake between 25% and 30% [30,31,32,33,35]

### 2.5. Training Protocol

Participants performed the 30-min Curves circuit exercise program (Curves International, Waco, TX, USA) three days per week over the course of the 14-week study. The circuit included 13 bi-directional hydraulic concentric-only resistance exercise machines which worked all major muscle groups (i.e., elbow flexion/extension, knee flexion/extension, shoulder press/latissimus dorsi pull, hip abductor/adductor, chest press/seated row, horizontal leg press, squat, abdominal crunch/back extension, chest flies, oblique, shoulder shrug/dip, hip extension, and side bends). During each training session, participants were coached to perform as many repetitions as possible within a 30-s time period on each resistance machine. Between machines, participants performed floor-based aerobic exercises or stepping exercise for 30 s with a goal of maintaining an elevated heart rate. We previously reported that women participating in this type of training elicit an average exercise heart rate of 126 ± 15 bpm (80% of maximal heart rate), an average exercise intensity of 65 ± 10% of peak oxygen uptake, resistance exercise intensities ranging between 61% and 82% of 1RM on the various exercise machines, and expenditure of 314 ± 102 kcal per workout [36,37,38]. Participants completed the entire circuit twice during each workout and then performed stretching exercises. All workouts were supervised by trained fitness instructors who monitored proper exercise technique and maintenance of adequate exercise intensity. Compliance to the exercise program was set a priori at a minimum of 70% compliance (30/42 exercise sessions). 

## 3. Procedures

### 3.1. Dietary Assessment

Participants were provided a detailed description of how to measure and record food and beverage intake by a registered dietitian prior to the start of the study. Participants recorded all food and energy containing fluids consumed for 4 day (including one weekend day) prior to each testing session. Dietary records were checked for accuracy prior to submission each testing session and analyzed by a registered dietitian using dietary analysis software (ESHA Food Processor Version 8.6, Salem, OR, USA).

### 3.2. Resting Energy Expenditure and Metabolism

Resting energy expenditure (REE) was assessed using a Parvo Medics TrueMax 2400 Metabolic Measurement System (ParvoMedics, Inc., Sandy, UT, USA). This test was performed in a fasted state with the participants lying supine on an exam table. A clear, hard plastic hood and a soft, clear plastic drape were placed over the participants’ neck and head in order to determine resting oxygen uptake and energy expenditure. All participants remained motionless without falling asleep for approximately 20 min. Data were recorded after the first ten minutes of testing during a five-minute period of time in which criterion variables (e.g., VO_2_ L/min) changed less than 5% [39] Test–retest measurements on 14 participants from a study previously reported [30] revealed that test–retest correlations (*r*) of collected VO_2_ in L/min ranged from 0.315 to 0.901 (mean 0.638) and the coefficient of variation ranged from 8.2 to 12.0% (mean: 9.9%) with a mean intra-class coefficient of 0.942, *p* < 0.001.

### 3.3. Anthropometric Measures and Body Composition

Height and body weight were measured according to standard procedures using a calibrated electronic scale (Cardinal Detecto Scale Model 8430, Webb City, MO, USA), with a precision of +/−0.02 kg. Waist and hip circumference was measured using a Golnick tensiometer using standard criteria [40]. Total body water was estimated using a Xitron 4200 Bioelectrical Impedance Analyzer (Xitron Technologies, Inc., San Diego, CA, USA) in order to monitor hydration status. Body composition and bone density (excluding the cranium) were evaluated using calibrated Hologic Discovery W (Hologic Inc., Waltham, MA, USA) dual energy X-ray absorptiometry (DEXA) equipped with APEX Software (APEX Corporation Software, Pittsburg, PA, USA). Test–retest reliability studies performed with this DEXA machine have previously yielded mean coefficients of variation for total bone mineral content and total fat free/soft tissue mass of 0.31–0.45% with a mean intra-class correlation of 0.985 [41]. 

### 3.4. Exercise and Functional Capacity

Resting heart rate was determined by palpation of the radial artery using standard procedures [40]. Blood pressure was assessed by auscultation of the brachial artery using an aneroid sphygmomanometer using standard clinical procedures [40]. Resting heart rate and blood pressure measurements were taken on the participant in the supine position after resting for 5 min. Participants were attached to a Quinton 710 ECG (Quinton Instruments, Bothell, WA, USA) and walked on a Trackmaster TMX425C treadmill (JAS Fitness Systems, Newton, KS, USA). Expired gases were collected using a Parvo Medics 2400 TrueMax Metabolic Measurement System (ParvoMedics, Inc., Sandy, UT, USA). Participants then performed a standard symptom-limited maximal Bruce treadmill exercise test according to standard procedures [40]. Calibration of gas and flow sensors was completed every morning prior to testing and was found to be within 3% of the previous calibration point. 

A 6-min minute walk test (6MWT) was conducted using standardized procedures including a flat surface distance of 100-feet with intervals marked by colored tape on the ground. Participants were told to walk as far as possible for six minutes without running or jogging. Participants were allowed to stop and rest during the test, but were instructed to resume walking as soon as possible. The number of laps and distance were recorded after 6 min. The test–retest reliability for the 6MWT has been reported to range from *r* = 0.95 to *r* = 0.97 [42,43]. 

A standard isotonic Olympic bench press (Nebula Fitness, Versailles, OH, USA) was used for the isotonic bench press testing. A 1RM testing procedure was performed using standard procedures with 2-min recovery between attempts [40]. Following 1RM testing, participants performed maximum number of repetitions at 80% of 1RM on bench press to determine upper body muscular endurance. Participants were then given five minutes of rest and then had their lower body 1RM maximal strength determined using a hip sled/leg press (Nebula Fitness, Versailles, OH, USA) and standard testing procedures with 2-min rest recovery between attempts [40]. Participants then performed maximum number of repetitions at 80% of hip sled/leg press 1RM to assess lower body muscular endurance. Test to test reliability of performing these strength tests in our lab has yielded low mean coefficients of variation (CV) and high reliability for the bench press (CV: 1.9%, intra-class *r* = 0.94) and hip sled/leg press (CV: 0.7%, intra-class *r* = 0.91). 

Measurements of balance and functional capacity were obtained using the Neurocom SmartEquitest^®^ (Neurocom International, Portland, OR, USA). Data were collected on postural balance and mobility utilizing the Sensory Organization Test (SOT), Limits of Stability (LOS), Step Up and Over (SUO), and Sit to Stand (STS) tests following standardized procedures [44]. Test-to-test reliability of performing these tests in women aged 65–75 has been reported to be *r* = 0.92 [45]. 

### 3.5. Blood Collection and Analysis

Fasted whole blood and serum samples were collected using standard phlebotomy techniques. Whole blood samples were analyzed for complete blood counts with percent differentials using an Abbott Cell Dyn 3500 (Abbott Laboratories, Abbott Park, IL, USA) automated hematology analyzer. Serum samples were analyzed for a complete metabolic panel using a calibrated Dade Behring Dimension RXL (Siemens AG, Munich, Germany) automated clinical chemistry analyzer. The coefficient of variation (CV) for the tests using this analyzer was similar to previously published data for these tests (range: 1.0 to 9.6%) [46]. Serum insulin, adiponectin, and leptin were determined using commercially available immuno-absorbent assay (ELISA) kits (Diagnostic Systems Laboratories, Webster, TX, USA) in conjunction with a Wallac Victor-1420 microplate reader (Perkin-Elmer Life Sciences, Boston, MA, USA) according to kit specifications. Intra-assay and inter-assay coefficients of variation were 4–7% for insulin, 3–4% for adiponectin, and 2–8% for leptin. The homeostasis model assessment for estimating insulin resistance (HOMA_IR_) was calculated as the product of fasting glucose multiplied by fasting insulin expressed in conventional units divided by 405 [47]. 

### 3.6. Statistical Analysis

Data were analyzed using IBM^®^ SPSS^®^ version 25 Statistics for Windows (IBM Corp., Armonk, NY, USA). Baseline variables were analyzed using one-way analysis of variance (ANOVA). Related variables were analyzed using univariate, multivariate and repeated measures general linear model (GLM) statistics. The overall multivariate Wilks’ Lamda time and group × time interaction *p*-levels were reported in tables along with Greenhouse–Geisser univariate tests, time and group × time effects, and between-subject group effects. Delta values (post—pre) as well as percent change from baseline values were calculated on select variables in order to normalize any baseline differences among groups and analyzed by one-way ANOVA with least significant difference (LSD) post-hoc analyses. Delta data are presented as mean changes from baseline with 95% confidence intervals (CIs). Mean changes with 95% CIs completely above or below baseline are considered significantly different [48]. Previous research in our lab demonstrated that an *n*-size of 20 per group was sufficiently powered to detect significant differences among diet groups in primary outcome variables [29,30,31,32,33,35] Data were considered significant when the probability of type I error was 0.05 or less and statistical trends toward significance if the *p*-level ranged between 0.05 and 0.10. If a significant interaction alpha level was observed, least significant difference (LSD) post-hoc analyses was performed to determine where significance was obtained. All data are represented as means ± standard deviations (SD) unless otherwise noted. 

## 4. Results

### 4.1. Participant Demographics

Table 1 presents participant demographics. One-way ANOVA revealed that there were no statistically significant differences among groups at baseline for descriptive characteristics. Participants were aged 65.9 ± 4.6 years (range 60–75), 161 ± 5 cm tall, weighed 78.6 ± 10.6 kg with 43.5 ± 3.6% body fat, and had a body mass index of 30.5 ± 4.1 kg/m^2^. 

### 4.2. Energy and Macronutrient Intake

Table 2 presents energy and macronutrient intake observed among groups throughout the study. Significant overall multivariate interaction effects (*p* < 0.001) were observed among energy intake and macronutrient intake expressed in absolute and relative terms. Univariate analysis of energy intake absolute values revealed that energy intake averaged 1482 ± 41 kcal/d (mean ± standard error of mean (SEM)) during the study with no significant interaction effects observed among groups. However, participants in the HP group ingested significantly more protein (*p* = 0.002) and less carbohydrate (*p* = 0.017) during the active weight loss portion of the diet (Phase II) compared to those in the HC and E groups. A similar pattern was observed when expressing energy and macronutrient intake relative to body weight. In this regard, adherence to the HP diet resulted in a 54% increase in relative protein intake (0.83 ± 0.16 to 1.28 ± 0.54 g/kg/d) and a 23% decrease in relative carbohydrate intake (2.50 ± 0.50 to 1.92 ± 0.56 g/kg/d) during the active weight loss phase. No significant interaction effects were observed among groups in dietary fat intake. Participants did a fairly good job meeting energy intake goals during Phase II of the diet (i.e., 1600 kcal/d). However, similar to our previous reports [29,30,31,32,33,34,35], participants were less successful increasing energy intake during the weight maintenance (Phase III) portion of the diet (i.e., 2100 kcal/d). 

### 4.3. Anthropometrics, Body Composition, and Resting Energy Expenditure

**Figure 3:**
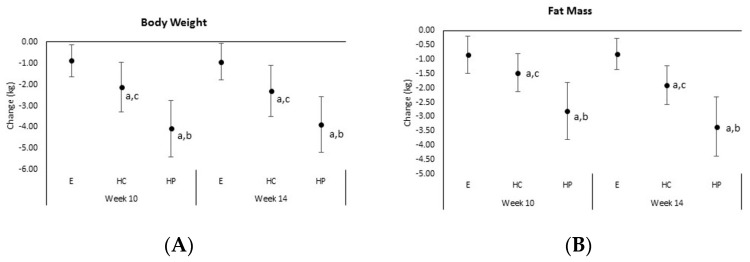

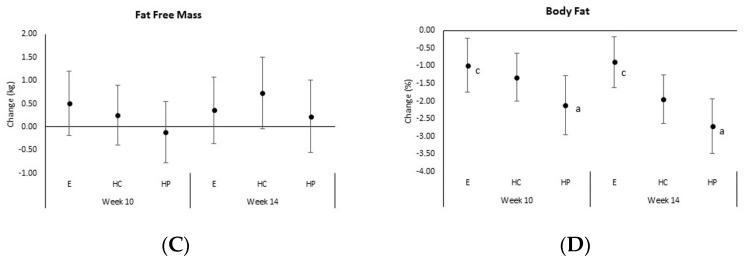
(**A**) Body weight, (**B**) fat mass, (**C**) fat-free mass, (**D**) body fat. Mean changes with 95% confidence intervals (CIs) from baseline in body composition variables for the exercise only (E), higher carbohydrate (HC), and higher protein (HP) groups. Means and 95% CIs completely below baseline represent a significant change over time. a = *p* < 0.05 difference from E; b = *p* < 0.05 difference from HC; c = *p* < 0.05 difference from HP.

Table 3 presents body composition, anthropometric, and resting energy expenditure data observed among groups during the study. A significant overall multivariate interaction effect (*p* < 0.001) was observed for body composition variables. Univariate analysis revealed significant interaction effects (*p* < 0.005) were observed among groups in body weight, fat mass, and percent body fat. Analysis of mean changes from baseline with 95% CI (Figure 3) indicated that participants in the HP group lost significantly more weight (E −0.87 ± 1.56, CI −1.63, −0.12; HC –2.12 ± 2.36, CI −3.30, −0.95; HP −4.07 ± 2.59, CI −5.4, −2.7 kg, *p* < 0.001) and fat mass (E −0.82 ± 1.12, CI −1.36, −0.28; HC −1.90 ± 1.38 CI −2.59, −1.21; HP −3.35 ± 2.02, CI −4.39, −2.3; kg, *p* < 0.001) while changes in body fat percentage were greater in the HP compared to E groups (E −0.89 ± 1.49, CI −1.61, −0.18; HC −1.94 ± 1.39, CI −2.64, −1.25; HP −2.71 ± 1.51, CI −3.49, −1.93%, *p* = 0.002). In percentage terms, participants in the HP group experienced a more clinically impactful reduction in fat mass loss (E −2.7 ± 3.8, CI −4.6, −0.9; HC −5.9 ± 4.2, CI −8.0, −3.9; HP −10.2 ± 5.8, CI −13.1, −7.2%, *p* = 0.003) and percent body fat (E −2.0 ± 3.5, CI −3.7, −0.3; HC −4.3 ± 3.2, CI −5.9, −2.7; HP −6.3 ± 3.5, CI −8.1, −4.5%, *p* = 0.002) than those in the HC and E groups. Fat-free mass increased over time in all groups with no significant differences observed among groups (E 0.36 ± 1.49, CI −0.36, 1.07; HC 0.73 ± 1.55, CI −0.04, 1.49; HP 0.22 ± 1.51, CI −0.56, 1.00 kg, *p* = 0.596). An overall significant interaction was also observed among anthropometric measurements (*p* = 0.017) with waist and hip circumferences significantly decreasing from baseline. Analysis of mean changes with 95% CIs revealed that greater changes were observed in the HP group in waist circumference (E −0.90 ± 1.83, CI −1.78, −0.02; HC −1.55 ± 2.94, CI −3.01, −0.09; HP −3.62 ± 3.57, CI −5.46, −1.78 cm, *p* = 0.017) while changes in hip circumference tended to differ among groups (E −1.60 ± 2.42, CI −2.77, −0.43; HC −1.98 ± 349, CI −3.71, −0.24; HP −3.88 ± 2.86, CI −5.35, −2.42 cm, *p* = 0.057). When expressed as percent changes from baseline, participants in the HP group experienced significantly greater changes in waist circumference (E −1.0 ± 2.1, CI −2.0, 0.02; HC −1.6 ± 3.3, CI −0.3, 0.03; HP −3.9 ± 3.7, CI −5.8, −2.0%, *p* = 0.019). Resting energy expenditure did not significantly decrease over time in response to the diet and exercise intervention or differ among groups.

### 4.4. Hematological Markers

Table 4 presents fasting glucose homeostasis, appetite hormones, and lipid-related variables. Significant time effects (*p* < 0.001) were observed among glucose homeostasis variables with insulin and insulin sensitivity improving among all groups over time. However, no significant interactions were observed among groups. Mean change and 95% CI analysis revealed that fasting glucose decreased to a greater degree (*p* < 0.031) in the HP compared to the HC group (E −1.89 ± 10, CI −6.88, 3.10; HC 5.13 ± 16, CI –3.02, 13.3; HP −5.93 ± 17, CI −14.6, 2.8%, *p* = 0.089). Significant overall time (*p* < 0.001) and group × time (*p* < 0.001) effects were seen in appetite-related hormones. Specifically, post hoc analysis revealed that participants in the HP group experienced a significantly greater increase in adiponectin (E 43.1 ± 76, CI 6.34, 79.8; HC −27.8 ± 33, CI –44.5, −11.3; HP 52.4 ± 79, CI 11.9, 92.8%, *p* = 0.001) than the HC group and the HP group observed a greater reduction in leptin (E −1.80 ± 34, CI −18.1, 14.5; HC 43.8 ± 60, CI 16.5, 71.2; HP −26.5 ± 70, CI −62.6, 9.6%, *p* = 0.001) than the HC and E groups. In percentage terms, greater changes were seen in the HP group in adiponectin (E 43.1 ± 76.2, CI 6.3, 79.8; HC −27.9 ± 33.3, CI −44.4, −11.3; HP 52.4 ± 78.6, CI −11.9, 92.8%, *p* = 0.001) and leptin levels (E −1.80 ± 33.8, CI −18.1, 14.5; HC 43.8 ± 55.0, CI 16.5, 71.1; HP −26.5 ± 70.3, CI −62.6, 9.6%, *p* = 0.001). Finally, no significant overall time effects were seen in lipid-related variables (*p* = 0.124) while these variables tended to interact (*p* = 0.056). Univariate analysis revealed significant interactions in triglycerides among groups where values in the HC and E groups increased from baseline after 10 weeks, while remaining consistent in the HP group. Table S1 presents serum liver and muscle enzyme related panels. Overall multivariate time (*p* < 0.001) and interaction (*p* < 0.001) effects were observed in clinical markers of liver function. Univariate analysis revealed some variable changes over time and among groups in alanine amino transferase (ALT) and total bilirubin (TBIL) responses, but these values remained well-within normal limits and were generally lower than baseline values. No overall time (*p* = 0.274), or interaction effects (*p* = 0.122) were observed among serum protein and enzyme levels. Table S2 presents whole blood complete cell count data while Table S3 presents lymphocyte percent differentials. Although overall time effects were seen (*p <* 0.001) in whole blood white and red cell blood count analysis, no significant interactions were observed (*p* = 0.342). Moreover, changes, if any, were small and well within clinical norms for older individuals. No significant multivariate time (*p* = 0.394) or group × time (0.664) effects or univariate effects were seen in percentage of lymphocytes. 

### 4.5. Exercise and Functional Capacity

Table 5 depicts health and fitness-related variables. Multivariate analysis revealed that exercise training improved resting hemodynamics over time (*p* = 0.047) with no significant interaction effects (*p* = 0.662). Univariate analysis demonstrated that resting heart rate tended to decrease over time (*p* = 0.074) while resting diastolic blood pressure was significantly reduced from baseline over time (*p* = 0.032) with no significant interactions observed among groups in resting heart rate (*p* = 0.455), systolic blood pressure (*p* = 0.814), or diastolic blood pressure (*p* = 0.39) responses. Significant time effects (*p* < 0.001) were seen in peak aerobic capacity and 6-min walk test distance with no significant differences observed among groups. The improved distance observed in 6-min walk test performance (34.5 ± 39 m) exceeded the minimal clinically important differences reported in the literature of 14 m to 20 m [49,50] but were slightly below the substantial meaningful difference of 50 m [50]. Upper and lower 1RM strength and bench press muscular endurance were significantly increased in all groups over time with no differences observed among groups. Tables S4 and S5 present balance and functional stability test results, respectively. Composite scores on the sensory organization and the limits of stability test significantly improved over time in all groups. Additionally, there was evidence that lift up index movement time, rising index, and left to right weight symmetry improved with training in all groups. However, no significant interactions among groups were observed. 

## 5. Discussion and Conclusions

Aging is associated with an increase in adiposity in sedentary individuals that increases the prevalence of obesity and/or severity of comorbidities associated with obesity such as insulin resistance and diabetes [1]. Sedentary lifestyle is also associated with loss of muscle mass and strength which increases risk to falls and bone fracture as one ages, particularly in osteoporotic women [7,18,21]. Therefore, identifying strategies that can help overweight and/or obese older adults promote weight and fat loss while maintaining muscle mass and strength may help reduce risk to obesity, age-related comorbidities, and/or injuries. We have previously reported that adherence to a circuit-style resistance exercise program and a higher protein low fat diet promoted greater weight and fat loss while maintaining muscle mass and preserving resting energy expenditure to a greater degree in comparison to following a traditional higher carbohydrate low fat diet in women under the age of 55 [29,30,31,32,33,34,35]. Moreover, this program promoted gains in aerobic capacity, muscular strength and endurance, and improved markers of health and metabolic syndrome [29,30,31,32,33,34,35]. We hypothesized that incorporating resistance exercise while maintaining a higher protein hypo-energetic diet may promote more favorable changes in body composition in older individuals attempting to lose weight and fat mass compared to adherence to a higher carbohydrate diet and/or exercise alone. Results of this study indicate that this exercise and diet approach may be an effective way for older sedentary and overweight women to achieve meaningful weight loss and improve markers of health and functional capacity. 

### 5.1. Weight Loss

Obesity is a significant health issue particularly as one ages [1]. However, diets that are too energy-restrictive promote weight loss at the expense of loss in muscle mass and/or reductions in resting energy expenditure [1,49]. Given concerns over sarcopenia and loss of strength as one ages, use of traditional energy deficit diet interventions may therefore exacerbate these conditions. Resistance exercise and increasing dietary protein have been recommended as a means of maintaining muscle mass and strength as one ages [17,25,27,50]. The reason for this is that resistance exercise stimulates muscle protein synthesis which can help increase and/or maintain muscle mass [51]. Additionally, digestion of dietary protein is less efficient as one ages, thereby increasing dietary protein needs [28]. There is also evidence that higher protein diets promote satiety, influence appetite hormones, and associated with a greater energy expenditure due to an increased thermic of digesting and oxidizing proteins, and improve glucose homeostasis [52]. 

Results from the present study indicate that adherence to a resistance-based exercise program promoted modest improvements in weight loss and fat loss while maintaining fat free mass and resting energy expenditure. Thus, encouraging older individuals to incorporate resistance exercise into their exercise program can help maintain muscle mass. Further, participants consuming a hypo-energetic diet that contained a higher amount of dietary protein experienced greater loss of weight and fat mass than those ingesting a standard higher carbohydrate and low fat diet. In percentage terms, participants in the HP group experienced a more clinically impactful reduction in fat mass loss and percent body fat than those in the HC and E groups. Moreover, fat-free mass increased in all groups and REE was well-maintained. Thus, this program promoted effective weight loss (i.e., fat loss) without a loss of fat free mass or resting energy expenditure that is often associated with dieting. It should be noted that the diet intervention used only increased daily protein intake from 0.83±0.16 to 1.28 ± 0.54 g/kg/d. Therefore, although we called this a higher protein diet, this only represented a modest increase in dietary protein intake above the Recommended Daily Allowance in the United States (i.e., 0.8 g/kg/d) and within recommendations for older adults (i.e., 1–1.3 g/kg/d) [27,49] as well as active individuals who engage in resistance training [53,54]. It is also interesting to note that fat-free mass and resting energy expenditure were maintained in the HC, suggesting that the participants ingested enough protein in their diet, in combination with the resistance exercise, to prevent the typical decline in energy expenditure and muscle mass associated with weight loss.

The present study findings are also consistent with our prior reports that adherence to the circuit-style resistance exercise program and higher protein diet promotes greater fat loss while maintaining muscle mass and resting energy expenditure in women under the age of 55 [29,30,31,32,33,34,35]. Similarly, but without exercise, energy restricted high protein diet (35%) compared to an energy-restricted diet with lower protein (20%) resulted in greater fat loss and improvements in cardio-metabolic profile variables in overweight and obese women (44 ± 9 years) [55]. These observations of improved body composition have also been noted in older men (65 ± 5 years) increasing their dietary protein intake from 97 ± 79 to 164 ± 89 g/d (1.26 to 2.12 g/kg/d) without caloric restriction compared to controls [20]. Finally, a report from Lee and colleagues [56] showed that adherence to a higher protein/low fat hypo-energetic diet (32% protein, 12% fat) was more efficacious in promoting fat loss than adherence to a lower protein hypo-energetic diet (22% protein, 12% fat) in overweight men and women.

### 5.2. Markers of Health

Results of the present study indicated that participation in this exercise and diet intervention promoted general improvements in a number of markers of health. For example, significant time effects were seen in waist circumference (2.1 ± 3.3%), hip circumference (−2.2 ± 2.7%), resting heart rate (−2.5 ± 15%), resting diastolic blood pressure (−3.4 ± 15%), fasting insulin (93.7 ± 140%), HOMA_IR_ (91.8 ± 137%), and adiponectin (22.3 ± 74.1%) levels. Participants in the HP group experienced significantly greater changes in waist circumference, blood glucose, adiponectin, and leptin levels. Moreover, no clinically significant changes were seen in clinical blood profiles. These findings are consistent with our prior reports in younger women [29,30,31,32,33,34,35]. Additionally, they are consistent with reports indicating that exercise and/or diet-induced weight loss improves markers of health [20,29,30,31,32,33,34,35,45,55], insulin sensitivity [4,20,29,31,32,35,55,57,58,59,60,61,62], and can positively affect appetite hormone regulation [30,31,32,35,57,58,63,64]. Moreover, these findings are consistent with reports that replacing some dietary carbohydrate with protein in the diet may promote greater benefits for weight loss, body composition, blood lipids, and/or blood glucose management. Results also support contentions that this exercise and diet intervention is well-tolerated in healthy older women.

### 5.3. Functional Capacity

One of the goals of encouraging older individuals to exercise is to maintain cardiovascular and musculoskeletal health in order to reduce risk to chronic disease, falls, and injury [20,21,22,24]. Results of the present study indicate that this exercise program was effective in improving markers of fitness, strength, and functional capacity. In this regard, participants experienced a 10.8 ± 23.9% improvement in peak aerobic capacity, a 7.2 ± 8.9% increase in distance completed during a 6-min walk test, a 22.5 ± 26.7% increase in upper extremity maximal strength, a 39.9 ± 75.6% increase in upper extremity muscular endurance, a 33.6 ± 37.2% increase in low extremity maximal strength, and a 27.1 ± 65.9% increase in lower extremity muscular endurance. The improved distance observed in 6-min walk test performance (34.5 ± 39 m) exceeded the minimal clinically important differences reported in the literature of 14 m to 20 m [42,43] but were slightly below the substantial meaningful difference of 50 m [42]. Moreover, significant improvements were observed in several balance-related functional capacity tests. These findings support contentions that older individuals can significantly benefit from participation in a structured exercise program and this could play an important role in reducing risk to falls and injury [19,20,21,22,45,65]. However, increasing the proportion of dietary protein during the diet phase did not promote greater improvement in fitness, strength, and/or functional capacity than those in the HC or E groups.

### 5.4. Conclusions

Results from this study indicate that older but otherwise healthy women following a higher protein diet while participating in a circuit-style resistance-exercise program experienced greater loss in body weight, fat mass, percent body fat, and waist circumference than women following a higher carbohydrate weight loss diet. Additionally, weight loss can be achieved in this population without significant reductions in fat-free mass or resting energy expenditure whether exercising alone or following the HC or HP diets. Participants in the HP group also experienced better management of blood glucose and appetite-related hormones, while all participants experienced improvement in a number of health, fitness, and functional capacity related variables. Results support recommendations that older individuals participate in resistance-training [4,19,20,22,50,53,54,66,67] and consume a higher proportion of protein in their diet in order to maintain muscle mass [4,26,27,28,53]. However, future research is needed to confirm results in a larger population of healthy older individuals as well as individuals who have medical conditions in which exercise and/or weight loss may provide benefits. Additionally, it is necessary to continue to investigate the effects of different diet and exercise strategies on markers of health in older individuals in order to identify effective strategies to reduce risk of and/or manage chronic disease and improve quality of life.

## Figures and Tables

**Table 1:** Baseline demographics.

Variable	E	HC	HP	*p*-Value
Age (years)	66.0 ± 4.3	63.3 ± 4.8	65.5 ± 5.2	0.87
Weight (kg)	76.0 ± 9.5	78.6 ± 10.7	81.6 ± 11.4	0.29
Height (cm)	159.6 ± 3.7	161.3 ± 4.9	161.7 ± 6.8	0.41
BMI (kg/m^2^)	29.9 ± 4.1	30.3 ± 4.0	31.3 ± 4.2	0.61
Body fat (%)	42.7 ± 3.7	44.2 ± 3.5	43.6 ± 3.7	0.46

Data are expressed as means ± standard deviations for the exercise only (E, *n* = 19), high carbohydrate (HC, *n* = 18), and high protein (HP, *n* = 17) groups. BMI: body mass index.

**Table 2:** Energy and macronutrient intake.

Variable	Group	Week			Group (SEM)	Source	*p*-Value
		0	10	14			
Energy intake	E	1540 ± 397	1580 ± 399	1490 ± 485	1537 ± 70	Group	0.004
(kcal/d)	HC	1339 ± 321	1253 ± 269	1260 ± 308	1283 ± 70 ^c^	Time	0.854
	HP	1598 ± 415	1567 ± 292	1711 ± 352	1626 ± 74 ^b^	G × T	0.322
	Mean	1489 ± 388	1462 ± 355	1478 ± 425			
Protein	E	68.3 ± 13.9	73.7 ± 11.0 ^bc^	68.5 ± 17.0 ^c^	70.2 ± 2.5	Group	<0.001
(g/d)	HC	57.1 ± 13.5	55.3 ± 9.5 ^ac^	61.6 ± 16.1 ^a^	58.0 ± 2.5 ^c^	Time	0.003
	HP	66.1 ± 13.0	94.9 ± 31.4 †^ab^	86.2 ± 18.3 †^ab^	82.4 ± 2.6 ^b^	G × T	0.002
	Mean	63.7 ± 14.1	73.9 ± 24.9 †	71.6 ± 19.6 †			
Carbohydrate	E	196 ± 70	182 ± 61 ^c^	173 ± 62	187 ± 11	Group	0.290
(g/d)	HC	166 ± 52 ^c^	171 ± 56	168 ± 51	163 ± 11	Time	0.022
	HP	203 ± 56 ^b^	146 ± 44 †^a^	193 ± 67	181 ± 12	G × T	0.017
	Mean	188 ± 61	173 ± 54 †	182 ± 60			
Total fat	E	58.6 ± 20.1	65.7 ± 19.4 ^b^	67.4 ± 22.5 ^b^	63.9 ± 3.7 ^b^	Group	0.002
(g/d)	HC	48.9 ± 12.5	43.6 ± 9.9 ^ac^	43.4 ± 14.9 ^ac^	45.3 ± 3.5 ^ac^	Time	0.415
	HP	54.9 ± 15.4	63.0 ± 21.9 ^b^	58.7 ± 23.4 ^b^	58.8 ± 3.5 ^b^	G × T	0.101
	Mean	53.9 ± 16.3	57.1 ± 20.1	56.1 ± 22.5			
Calories	E	20.1 ± 6.0	21.5 ± 5.5	20.4 ± 7.0	20.9 ± 1.06	Group	0.015
(kcal/kg/d)	HC	17.3 ± 4.5 ^a^	16.8 ± 4.4 ^ac^	17.0 ± 5.2 ^c^	17.0 ± 1.06 ^ac^	Time	0.550
	HP	19.8 ± 4.4	20.8 ± 4.6	22.7 ± 5.4	21.1 ± 1.12	G × T	0.234
	Mean	19.3 ± 5.2	19.6 ± 5.2	19.9 ± 6.3			
Protein	E	0.93 ± 0.24	1.01 ± 0.22	0.94 ± 0.27	0.96 ± 0.05	Group	<0.001
(g/kg/d)	HC	0.74 ± 0.19 ^a^	0.73 ± 0.15	0.83 ± 0.26	0.77 ± 0.04	Time	<0.001
	HP	0.83 ± 0.16	1.28 ± 0.5 ^ab^	1.15 ± 0.30 ^b^	1.09 ± 0.05 ^ab^	G × T	0.001
	Mean	0.83 ± 0.21	1.00 ± 0.40 †	0.97 ± 0.30 †			
Carbohydrate	E	2.66 ± 1.1	2.56 ± 0.9 ^c^	2.47 ± 1.0	2.56 ± 0.16	Group	0.233
(g/kg/d)	HC	2.14 ± 07	2.21 ± 0.8	2.14 ± 0.7	2.16 ± 0.16	Time	0.127
	HP	2.50 ± 0.5	1.92 ± 0.5 ^a^	2.54 ± 0.9	2.32 ± 0.17	G × T	0.029
	Mean	2.43 ± 0.80	2.24 ± 0.8	2.38 ± 0.9			
Total fat	E	0.74 ± 0.23	0.85 ± 0.27	0.80 ± 0.32	0.79 ± 0.05	Group	0.004
(g/kg/d)	HC	0.63 ± 0.18	0.58 ± 0.14	0.59 ± 0.23	0.60 ± 0.05 ^ac^	Time	0.100
	HP	0.73 ± 0.24	0.87 ± 0.30	0.90 ± 0.34	0.83 ± 0.05	G × T	0.072
	Mean	0.70 ± 0.22	0.76 ± 0.28	0.76 ± 0.32			

Data are expressed as means ± standard deviations for the exercise only (E, *n* = 19), high carbohydrate (HC, *n* = 18)) and high protein (HP, *n* = 17) groups. General linear model analysis of absolute values revealed overall Wilks’ Lambda time (*p* = 0.002) and group × time (*p* = 0.001) effects. General linear model analysis of relative diet intake values revealed overall Wilks’ Lambda time (*p* = 0.002) and group × time (*p* = 0.002) effects. Greenhouse–Geisser univariate *p*-levels are listed for group (G), time (T), and group × time (G × T) interaction effects. † represents *p* < 0.05 difference from baseline value. ^a^ = *p* < 0.05 difference from E; ^b^ = *p* < 0.05 difference from HC; ^c^ = *p* < 0.05 difference from HP.

**Table 3:** Body composition, anthropometric, and resting energy expenditure data.

Variable	Group	Weeks			Group (SEM)	Source	*p*-Value
		0	10	14			
Body weight	E	75.99 ± 9.5	75.11 ± 9.7	75.12 ± 9.7	75.39 ± 2.4	Group	0.606
(kg)	HC	78.63 ± 10.7	75.50 ± 11.1 †	76.50 ± 11.1 †	77.15 ± 2.5	Time	<0.001
	HP	81.59 ± 11.4	77.63 ± 10.8 †	77.71 ± 10.8 †	78.94 ± 2.6	G × T	<0.001
	Mean	78.63 ± 10.6	76.34 ± 10.4 †	78.34 ± 10.4			
Fat mass	E	29.89 ± 6.2	29.05 ± 6.0 †	29.07 ± 6.1 †	29.34 ± 1.4	Group	0.772
(kg)	HC	31.89 ± 6.7	30.43 ± 6.6 †	29.99 ± 6.5 †	30.77 ± 1.4	Time	<0.001
	HP	32.78 ± 6.0	29.98 ± 5.7 †	29.42 ± 5.6 †	30.73 ± 1.5	G × T	<0.001
	Mean	31.46 ± 6.3	29.80 ± 6.0 †	29.49 ± 6.0 †			
Fat free mass	E	39.66 ± 4.1	40.1 ± 4.2	40.01 ± 4.3	39.95 ± 1.2	Group	0.351
(kg)	HC	39.86 ± 4.6	40.1 ± 4.9	40.59 ± 5.4	40.19 ± 1.2	Time	0.064
	HP	42.40 ± 5.8	42.09 ± 6.1	42.42 ± 6.6	42.23 ± 1.3	G × T	0.427
	Mean	40.53 ± 4.9	40.75 ± 5.1	40.96 ± 5.5			
Body fat	E	42.68 ± 3.7	41.69 ± 3.7 †	41.78 ± 3.5 †	42.05 ± 0.8	Group	0.584
(%)	HC	44.17 ± 3.5	42.85 ± 3.5 †	42.23 ± 3.3 †	43.08 ± 0.8	Time	<0.001
	HP	43.57 ± 3.8	41.45 ± 3.8 †	40.86 ± 4.3 †	41.96 ± 0.9	G × T	0.005
	Mean	43.46 ± 3.6	42.00 ± 3.6 †	41.63 ± 3.7 †			
Bone mineral	E	1531 ± 183	1527 ± 179	1535 ± 182	1531 ± 68 ^a^	Group	0.045
content (g)	HC	1613 ± 214	1615 ± 212	1608 ± 227	1612 ± 70	Time	0.349
	HP	1789 ± 427	1767 ± 454	1788 ± 439	1781 ± 72 ^c^	G × T	0.365
	Mean	1640 ± 305	1632 ± 312	1639 ± 312			
Waist	E	88.4 ± 7.9	88.3 ± 7.3	87.5 ± 7.6	88.1 ± 2.2	Group	0.978
circumference	HC	89.7 ± 12.9	88.9 ± 12.0	88.2 ± 12.1	89.9 ± 2.2	Time	<0.001
(cm)	HP	91.2 ± 8.2	89.1 ± 7.5 †	87.6± 7.6 †	89.3 ± 2.3	G × T	0.016
	Mean	89.7 ± 9.0	88.7 ± 9.0 †	87.8 ± 9.2 †			
Hip	E	107.6 ± 8.1	107.0 ± 8.1	106.0 ± 7.4	106.9 ± 1.7	Group	<0.001
circumference	HC	109.9 ± 5.5	108.0± 6.3	107.9 ± 7.6 †	108.6 ± 1.7	Time	<0.001
(cm)	HP	112.7 ± 8.8	109.4 ± 8.2 †	108.8 ± 7.9 †	110.3 ± 18	G × T	0.019
	Mean	110.0± 7.5	108.1± 7.5 †	107.5 ± 7.6 †			
Waist:hip	E	0.822 ± 0.05	0.826 ± 0.08	0.827 ± 0.06	0.825 ± 0.01	Group	0.684
ratio	HC	0.815 ± 0.09	0.821 ± 0.08	0.815 ± 0.07	0.817 ± 0.02	Time	0.180
	HP	0.810 ± 0.05	0.815 ± 0.05	0.805 ± 0.05	0.810 ± 0.02	G × T	0.616
	Mean	0.816 ± 0.07	0.821 ± 0.06	0.816 ± 0.06			
Resting energy expenditure (kcal/d)	E	1317 ± 184	1341 ± 198	1345 ± 168	1334 ± 39	Group	0.923
	HC	1313 ± 199	1358 ± 211	1356 ± 181	1342 ± 37	Time	0.065
	HP	1311 ± 151	1361 ±130	1287 ± 176	1320 ± 43	G × T	0.356
	Mean	1314 ± 178	1353 ± 183	1332 ± 174			

Data are expressed as means ± standard deviations for the exercise only (E, *n* = 19), high carbohydrate (HC, *n* = 18)), and high protein (HP, *n* = 17) groups. General linear model analysis revealed overall Wilks’ Lambda time (*p* < 0.001) and group × time (*p* < 0.001) effects for body composition variables and overall Wilks’ Lambda time (*p* < 0.001) and group × time (*p* < 0.017) effects for anthropometric measurements. Greenhouse–Geisser univariate *p*-levels are listed for group (G), time (T) and group × time (G × T) interaction effects. † represents *p* < 0.05 difference from baseline value. ^a^ = *p* < 0.05 difference from E; ^c^ = *p* < 0.05 difference from HP.

**Table 4:** Fasting serum hormone and metabolic markers.

Variable	Group	Weeks			Group (SEM)	Source	*p*-Value
		0	10	14			
Glucose	E	109.0 ± 21	108.3 ± 23	106.5 ± 21	107.9 ± 5	Group	0.400
(mg/dL)	HC	96.8 ± 14	98.4 ± 9	100.2 ± 10	98.5 ± 5	Time	0.335
	HP	114.5 ± 61	105.3 ± 26	99.5 ± 19	106.4 ± 5	G × T	0.295
	Mean	103.7 ± 37	104.1 ± 21	102.2 ± 18			
Insulin	E	11.1 ± 6.5	14.0 ± 8.0	17.1 ± 9.5	14.1 ± 1.9	Group	0.459
(µU/mL)	HC	10.1 ± 6.5	9.7 ± 5.9	16.1 ± 7.7	12.0 ± 1.9	Time	<0.001
	HP	12.7 ± 12.8	15.3 ± 10.9	18.1 ± 12.3	15.4 ± 2.0	G × T	0.486
	Mean	11.3 ± 8.8	13.0 ± 8.0	17.1 ± 9.8 †			
Insulin sensitivity	E	3.17 ± 2.4	3.80 ± 4.5	4.61 ± 2.8	3.86 ± 0.6	Group	0.308
(HOMA_IR_)	HC	2.47 ± 1.6	2.39 ± 1.5	3.96 ± 1.9	2.94 ± 0.6	Time	<0.001
	HP	3.67 ± 3.8	4.46 ± 4.4	4.86 ± 4.3	4.33 ± 0.7	G × T	0.527
	Mean	3.10 ± 2.7	3.53 ± 3.1	4.47 ± 3.1 †			
Adiponectin	E	11.5 ± 5.7	10.3 ± 3.9	16.6 ± 14.2	12.8 ± 1.8	Group	0.844
(µg/mL)	HC	13.7 ± 6.1	13.1 ± 4.8	9.5 ± 5.1 ^a^	12.1 ± 1.8	Time	0.007
	HP	14.0 ± 7.7	6.5 ± 2.6 †	23.1 ± 25.1 †^b^	14.6 ± 1.9	G × T	0.002
	Mean	13.1 ± 6.5	10.1 ± 4.7 †	16.3 ± 17.3 †			
Leptin	E	36.7 ± 13.4 ^c^	31.3 ± 23.2	35.2 ± 17.5 ^b^	34.4 ± 3.5	Group	0.218
(ng/mL)	HC	36.7 ± 16.4 ^c^	32.8 ± 19.5	50.4 ± 24.6 †^ac^	39.9 ± 3.6	Time	<0.001
	HP	61.5 ± 18.6 ^ab^	37.9 ± 14.5 †	38.4 ± 19.7 †^b^	45.9 ± 3.7	G × T	<0.001
	Mean	44.5 ± 19.7	33.9 ± 19.4 †	41.3 ± 21.4			
Triglycerides	E	141 ± 95	172 ± 125 †^c^	138 ± 89 ^bc^	150 ± 19	Group	0.898
(mg/dL)	HC	145 ± 65	160 ± 78 †	158 ± 66 ^c^	154 ± 19	Time	0.186
	HP	159 ± 81	149 ± 67 ^a^	157 ± 84 ^a^	155 ± 20	G × T	0.046
	Mean	148 ± 80	161 ± 79	151 ± 79			
Total cholesterol	E	200 ± 36	215 ± 36	191 ± 31	202 ± 7	Group	0.343
(mg/dL)	HC	199 ± 64	201 ± 27	199 ± 31	199 ± 7	Time	0.062
	HP	214 ± 32	216 ± 28	204 ± 24	211 ± 7	G × T	0.470
	Mean	204 ± 46	210 ± 31	198 ± 29			
Low density	E	115 ± 35	127 ± 28	115 ± 32	119 ± 6	Group	0.455
lipoprotein	HC	114 ± 43	110 ± 21	109 ± 24	111 ± 6	Time	0.828
(mg/dL)	HP	122 ± 25	123 ± 24	120 ± 25	121 ± 6	G × T	0.594
	Mean	117 ± 35	120 ± 25	115 ± 27			
High density	E	54.1 ± 15.4	55.6 ± 16.2	55.3 ± 15.4	55.0 ± 3.0	Group	0.876
lipoprotein	HC	55.06 ± 10.4	55.1 ± 11.2	57.3 ± 13.1	55.8 ± 3.1	Time	0.239
(mg/dL)	HP	57.8 ± 18.9	55.5 ± 13.8	55.6 ± 12.9	56.3 ± 3.2	G × T	0.216
	Mean	55.6 ± 15.0	55.4 ± 13.7	56.1 ± 13.7			

Data are expressed as means ± standard deviations for the exercise only (E, *n* = 19), high carbohydrate (HC, *n* = 18)) and high protein (HP, *n* = 17) groups. General linear model analysis revealed overall Wilks’ Lambda time (*p* < 0.001) and group × time (*p* < 0.308) effects for glucose and insulin-related variables; an overall Wilks’ Lambda time (*p* < 0.001) and group × time (*p* < 0.001) effects for appetite related variables; and, an overall Wilks’ Lambda time (*p* = 0.124) and group × time (*p* < 0.056) effects for lipid related variables. Greenhouse–Geisser univariate *p*-levels are listed for group (G), time (T) and group × time (G × T) interaction effects. HOMA_IR_ = homeostatic model assessment for insulin resistance. † represents *p* < 0.05 difference from baseline value. ^a^ = *p* < 0.05 difference from E; ^b^ = *p* < 0.05 difference from HC; ^c^ = *p* < 0.05 difference from HP.

**Table 5:** Cardiovascular, aerobic capacity, and muscular strength and endurance measures.

Variable	Group	Weeks		Group (SEM)	Source	*p*-Value
		0	14			
Resting heart rate	E	77.4 ± 12.6	72.0 ± 9.3	74.7 ± 2.4	Group	0.283
(bpm)	HC	74.4 ± 12.8	73.9 ± 11.8	74.2 ± 2.4	Time	0.074
	HP	74.1 ± 11.4	71.4 ± 11.6	72.7 ± 2.6	G × T	0.455
	Time	75.4 ± 12.6	72.4 ± 10.8			
Resting systolic	E	125.7 ± 10.5	122.8 ± 13.2	124.3 ± 2.3	Group	0.038
blood pressure	HC	124.6 ± 12.5	123.7 ± 14.1	124.18 ± 23	Time	0.238
(mmHg)	HP	126.5 ± 16.3	121.9 ± 11.3	124.2 ± 2.4	G × T	0.814
	Time	125.6 ± 13.0	122.8 ± 12.8			
Resting diastolic	E	77.8± 9.80	71.9 ± 7.8	74.8 ± 1.6	Group	0.330
blood pressure	HC	73.3 ± 10.4	72.8 ± 9.4	73.1 ± 1.7	Time	0.032
(mmHg)	HP	75.1 ± 9.5	70.9 ± 7.5	73.0 ± 1.7	G × T	0.379
	Time	75.4 ± 9.9	71.9 ± 8.1 †			
Peak VO_2_	E	17.70 ± 3.3	18.39 ± 3.2	18.05 ± 0.6	Group	0.110
(mL/kg/min)	HC	15.53 ± 2.2	17.14 ± 2.8	16.34 ± 0.7	Time	<0.001
	HP	17.13 ± 3.7	19.29 ± 3.1	18.21 ± 0.7	G × T	0.227
	Time	16.82 ± 3.2	18.26 ± 3.2 †			
Walk test (6-min)	E	496 ± 67	534 ± 59	516 ± 13	Group	0.218
(m)	HC	517 ± 47	551 ± 40	533 ± 14	Time	<0.001
	HP	538 ± 70	570 ± 77	554 ± 14	G × T	0.860
	Time	516 ± 63	551 ± 61 †			
Bench press (1RM)	E	21.4 ± 5.0	26.2 ± 3.3	23.8 ± 1.2	Group	0.536
(kg)	HC	20.6 ± 5.6	23.4 ± 5.7	22.0 ± 1.2	Time	<0.001
	HP	20.6 ± 6.2	24.5 ± 4.5	22.5 ± 1.3	G × T	0.245
	Time	20.9 ± 5.5	24.7 ± 4.7 †			
Bench press	E	127.8 ± 62	143.4 ± 43	135.6 ± 10	Group	0.237
endurance volume	HC	98.5 ± 53	135.7 ± 58	117.1 ± 10	Time	<0.001
(kg)	HP	132.9 ± 43	148.0 ± 55	140.4 ± 11	G × T	0.568
	Time	119.0 ± 55	142.1 ± 52 †			
Leg press 1RM	E	89.4 ± 32	110.0 ± 34	99.7 ± 8	Group	0.781
(kg)	HC	82.7 ± 33	105.5± 37	94.1 ± 8	Time	0.025
	HP	78.1 ± 32	106.2 ± 28	92.2± 8	G × T	0.636
	Time	83.5 ± 32	107.2 ± 33†			
Leg press	E	936 ± 623	1182 ± 959	1059 ± 147	Group	0.792
endurance volume	HC	839 ± 451	1114 ± 708	977 ± 143	Time	0.122
(kg)	HP	1161 ± 732	1076 ± 375	1118 ± 152	G × T	0.229
	Time	972 ± 609	1125 ± 713			

Data are expressed as means ± standard deviations for the exercise only (E, *n* = 19), high carbohydrate (HC, *n* = 18)) and high protein (HP, *n* = 17) groups. General linear model analysis revealed overall Wilks’ Lambda time (*p* = 0.047) and group × time (*p* = 0.662) effects for resting hemodynamics, an overall Wilks’ Lambda time (*p* < 0.001) and group × time (*p* = 0.578) effects for aerobic exercise capacity variables, and an overall Wilks’ Lambda time (*p* < 0.001) and group × time (*p* = 0.152) effects for muscular strength and endurance-related variables. Greenhouse–Geisser univariate *p*-levels are listed for group (G), time (T), and group × time (G × T) interaction effects. † represents *p* < 0.05 difference from baseline value. 1RM: 1 repetition maximum. VO_2_: oxygen uptake.

## Supplementary Materials

The following are available online at http://www.mdpi.com/2072-6643/10/8/1070/s1, Table S1: Serum chemistry: liver and muscle enzyme-related variables, Table S2: Serum chemistry: complete blood counts, Table S3: Serum chemistry: lymphocyte percent differentials, Table S4: Sensory organization and limits of stability test variables, Table S5: Functional balance testing parameters.
